# Peace of mind: A quasi-experimental, mixed-method evaluation of a community-based mental health intervention for persons affected by Neglected Tropical Diseases

**DOI:** 10.1371/journal.pmen.0000423

**Published:** 2025-09-04

**Authors:** Maaike L. Seekles, Motto Nganda, Jacob Kadima, Lucas Sempe, Joy Kim, Pierre Omumbu, Junior Kukola, Stephanie M. Ngenyibungi, Florent Ngondu, Louis Sabuni, Laura Dean

**Affiliations:** 1 Department of International Public Health, Liverpool School of Tropical Medicine, Liverpool, Merseyside, United Kingdom; 2 The Leprosy Mission DRC, Kinshasa, Democratic Republic of Congo; 3 Institute for Global Health and Development Division, Queen Margarets University, Edinburgh, United Kingdom; 4 Programme Team, Effect:Hope, Markham, Canada; 5 Department of Psychology, University of Kinshasa, Kinshasa, Democratic Republic of Congo; 6 Leprosy Programme, Ministry of Health, Kinshasa, Democratic Republic of Congo; PLOS: Public Library of Science, UNITED KINGDOM OF GREAT BRITAIN AND NORTHERN IRELAND

## Abstract

Evidence consistently shows high levels of mental distress amongst populations affected by skin NTDs. Self-help groups are thought to be a key intervention strategy to support affected persons. However, to date, self-help interventions have largely been concerned with physical improvements as opposed to psychological outcomes. This paper provides an evaluation of the impact of the ‘Peace of Mind’ intervention in Kasai Province, DRC. Peace of Mind was a community-based, peer-led mental health intervention, combining lay counselling, mutual support, self-care, and income generation activities within a self-help group model to address health, psychosocial and economic impacts of skin-NTDs. This mixed-methods study used a quasi-experimental difference-in-difference approach. A survey measured levels of depression, anxiety, and stigma before and after intervention. To facilitate data matching, machine learning was used to predict (based on age, sex, health zone and disability status) which participants in the baseline would have attended the self-help groups. In addition, qualitative and participatory methods including photovoice, in-depth interviews and key informant interviews were completed to elicit the experiences of group members and health staff. Our findings show that after 6 months of intervention this holistic approach was effective at reducing levels of depression (PHQ-9 score reduction ranging on average from -3.5 to -6.7 points, *p* < .05) and anxiety (GAD-7 score reduction from -2.0 to -3.3 points, *p* < .05) in persons affected by skin NTDs. However, the prevalence of depression (64%) and anxiety (52%) remained high with suicidal thoughts reported by 35% of respondents at endline. Whilst we found no impact on stigma scores, qualitative data indicated improvements to self-esteem and ability to take part in community life. To our knowledge, this is the first study in the DRC to evaluate the impact of a community-based, peer-led intervention on mental health outcomes of persons affected by skin-NTDs. Holistic self-help groups have the potential to serve as a key component of integrated NTD/mental health service provision at community-level. However, this should be accompanied by the integration of stigma-reduction strategies, the strengthening of primary health care capacities, and the establishment of mental health services at secondary and tertiary care levels.

## Introduction

Neglected Tropical Diseases (NTDs) are a diverse set of 20 communicable and non-communicable diseases that impose a human, social, and economic burden on more than one billion people worldwide [1]. Skin NTDs are a sub-grouping of at least ten NTDs -including Leprosy, Lymphatic Filariasis, Buruli Ulcer and Onchocerciasis. Skin NTDs initially present with changes and lesions on the skin, and when left untreated can lead to irreversible, chronic symptoms including physical disability and permanent disfigurement [2–4]. Associated with poverty, skin NTDs are most endemic in low-and middle-income countries, where they often affect the poorest and most marginalised communities [5]. In combination with stigmatisation and discrimination, skin NTDs hamper the ability of many persons affected to complete livelihood activities and participate fully in society. Consequently, evidence consistently shows high levels of psychological co-morbidity amongst populations affected by skin NTDs [3,6–8].

The Democratic Republic of the Congo (DRC) has one of the highest burdens of NTDs across Africa, largely resultant from decades of conflict and precarious health infrastructure [9]. The Kasai Province, DRC was the setting of a violent conflict up until a few years ago. Between 2016 and 2019, fighting took place which at its height led to thousands of deaths and over a million people displaced. During this time, health infrastructure was weak and service provision for the treatment and management of NTDs scarce and frequently absent. The conflict has now largely subsided, however, widespread destruction and destitution remains, with schools and health centres destroyed, and further exacerbated pre-existing food insecurity and livelihood crises [10,11]. The Leprosy Mission (TLM) DRC is currently working in Kasai province and other areas of DRC to support persons affected by leprosy and other skin NTDs, but have identified a critical gap in being able to support mental health needs of persons affected. A recent exploratory study, undertaken by our study team, found high levels of mental health needs amongst persons affected by NTDs in the area. In total, 58.3% of men and 80.0% of women screened positive for major depressive disorder. Symptoms indicative of generalised anxiety disorder were displayed by 54.8% of men and 62.2% of women [8]. In addition, the study identified difficulties with practicing self-management routines, reduced socio-economic functioning and stigma as key issues for persons affected [12]. Professional psychosocial services are virtually non-existent in the area. In response, our mixed-method study aimed to co-develop and evaluate a community-based mental health intervention. The study builds on learnings from a holistic intervention in Nigeria that showed improvements in physical and psychosocial health of persons affected. Central to the success of the intervention in Nigeria was the use of participatory action research methods to co-design and implement the intervention [13]. We have underpinned our work by essential values of participatory health research, including: building on strengths and resources in the community; emphasising problems of local relevance; supporting systems development through cyclical processes; and prioritising co-learning and capacity strengthening amongst all partners [14].

### The Kasai “Ditalala Dia Moyo” (Peace of Mind) intervention

Our intervention was co-designed through participatory action research [14]. Phase one of the design involved working collaboratively with persons affected by skin-NTDs and health professionals to identify their challenges. Problem identification was completed in 2022 using participatory, qualitative and quantitative research methods as described in Nganda et al and Seekles et al [8,12]. Phase two brought together key stakeholders including the research team, members of TLM DRC, persons- affected and community, regional and national health workers and systems actors to collaboratively design the support group intervention.

**Fig 1:**
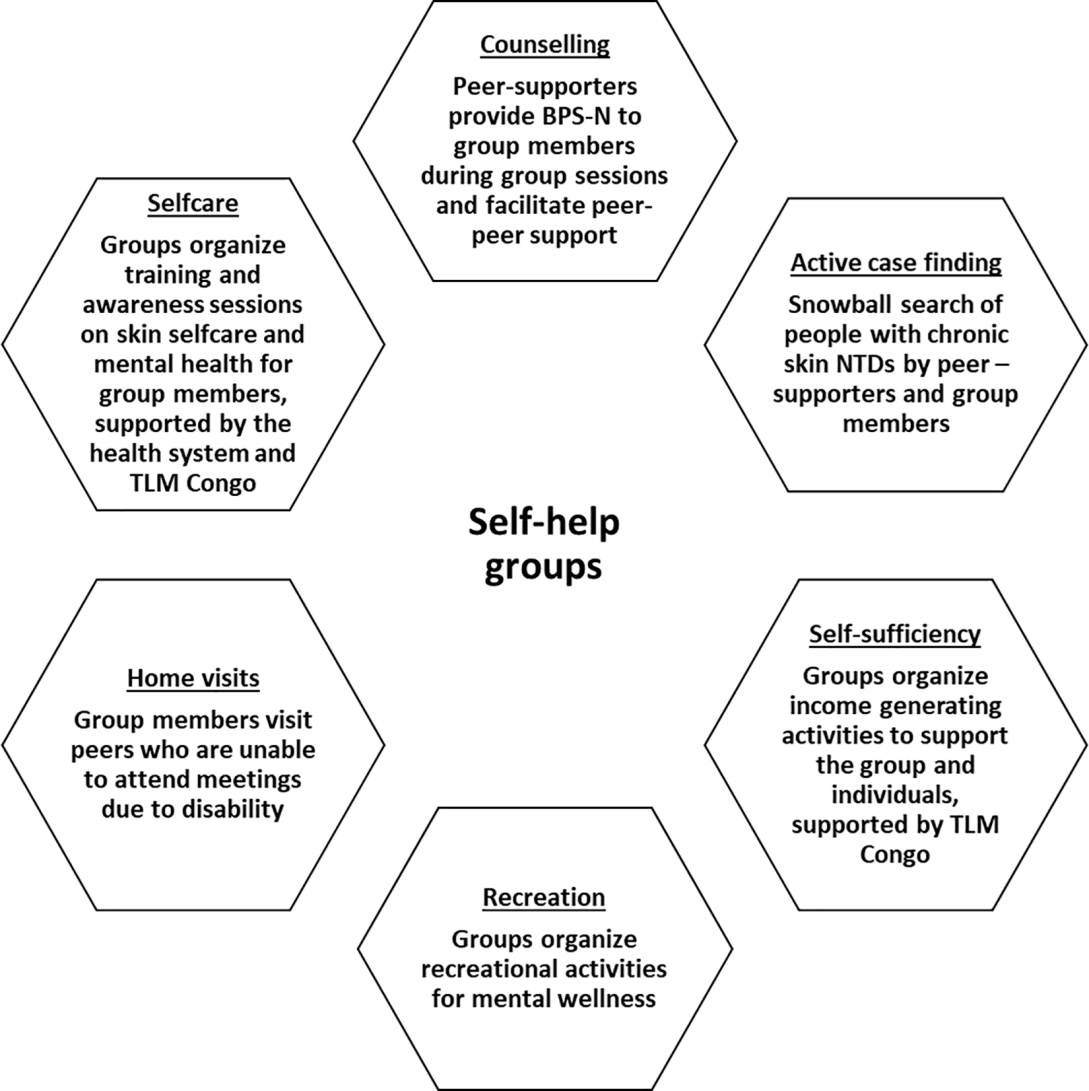
Self-help group core components (taken from Nganda et al [12]).

Through discussion, stakeholders agreed that the overall purpose of the intervention was to improve mental wellbeing of persons affected by NTDs through the provision of basic psychological support and strengthened peer-support networks. Using materials adapted by Agarwal et al [15,16] from the World Health Organisation’s ‘Psychological First Aid’ kit, within the Basic Psychological Support- NTDs (BPS-N), the intervention had four core components:

1. The provision of basic training for health workers across multiple health system levels (province, health zone, and health centre) on how to provide basic psychological support for NTDs (BPS-N).
2. The provision of basic training (using the BPS-N (adapted for context)) for community health workers and peer helpers (persons affected by NTDs) to support them to identify when persons affected by NTDs may be experiencing mental distress and need further support at the health centre.
3. To support community health workers (community relays) and peer helpers to establish self-help groups for persons affected by NTDs. Self-help groups were designed based on a combination of principles related to peer support and self-help groups. They were intended to be a safe space where persons affected could share common concerns, experiences and challenges, thereby creating a space for reciprocal physical and emotional support, in addition to undertaking income generating activities. Fig 1 provides an overview of the core-components of the group.
4. Ongoing support and supervision provided to trained health workers by The Leprosy Mission, health zone and provincial team.

Previous research has shown initial effectiveness of self-help groups for persons affected with skin-NTDs [15–17]. However these self-help groups have, so far, largely been concerned with physical (e.g., self-management/care, wound care) as opposed to psychological outcomes [18]. This paper provides an evaluation of the impact of the intervention on participant mental wellbeing, with an in-depth focus on self-help groups as a key component. Nganda & Seekles et al [19](forthcoming) provide further detail on the process of intervention delivery.

To our knowledge, this is one of few studies globally - and the first in the DRC – to evaluate the impact of a community-based, peer-led intervention on mental health problems (depression and anxiety) of persons affected by skin-NTDs.

## Materials and methods

### Study design and study sites

This study used a quasi-experimental pre/post intervention design, with a mixed-methods approach. The quantitative aspect included a survey, which measured levels of depression, anxiety, and stigma before and after intervention. In addition, qualitative and participatory methods including photovoice, in-depth interviews and key informant interviews were completed to elicit the experiences of self-help group members and health staff. The study was conducted in Ngombe (population: ~ 19,300) and Tshisele (population: ~ 13,600) health areas within Tshikapa Health Zone, Kasai Province, DRC. Four skin NTDs – Leprosy, Lymphatic Filariasis (LF), Buruli Ulcer (BU) and Onchocerciasis – are endemic in these areas [20,21] and were focused on in this study.

### Participants and recruitment

Any person over 18 years with diagnosed or self-reported NTD-related morbidity, including both visible and less-visible symptoms of Leprosy (e.g., shortening of extremities), LF (e.g., lymphoedema), BU, or Onchocerciasis (e.g., blindness) was eligible to participate in the self-help group intervention and associated evaluation activities.

To identify potential participants for the persons-affected survey, a census of persons-affected in both health areas was completed. Health centre records were searched and a process of active case finding and snowballing identified a total population of 125 affected persons across both health areas. A total of 118 persons affected completed the baseline survey. At endline, 19 persons had passed away or moved out of the area and 12 new persons affected had been identified during intervention roll-out. A total of 111 persons were targeted, of whom 99 successfully completed the follow-up survey. Of those, 58 had attended the self-help groups.

#### Self-help group establishment.

The establishment of the groups followed the processes described in the ‘Support Group Manual’, previously prepared by the COUNTDOWN programme in Nigeria [22]. The groups were led by peer leaders, who were trained by the research team and were called upon to perform a variety of tasks, including provision of one-directional support/basic counselling, outreach, advocacy and facilitation of meetings and group activities, including peer-peer support amongst group members. Recruitment into the groups was done by peer leaders, with support from community health workers (community relays), who would visit people with NTD symptoms and invite them to join. Persons affected could also self-select if they heard about the groups through other channels. In total, 60 persons-affected joined the self-help groups (29 in Ngombe and 31 in Tshisele), and 7 subgroups were formed (3 in Ngombe, and 4 in Tshisele). The groups met twice a month; not all persons affected joined every meeting. The focus of topics to be discussed in the groups was agreed by the group during their initial meetings and is further described in Table 4 (results).

### Data collection

#### Persons affected survey.

Baseline data collection was completed between the 7^th^ of February and 22^nd^ of April 2022. The follow-up study was conducted from April 3^rd^ until June 15^th^ 2023, six months after the start of the support groups. Surveys were completed by data collectors who were trained members of the local community. Data collectors were supported by the broader research team, including staff from TLM DRC (JK1, PPL, JK2) and LSTM (MS, LD, MN). Due to low literacy rates, data collectors described information sheets verbally in the presence of a witness. Informed consent was recorded, via signature or fingerprint. The survey was anonymous, administered verbally, and directly entered via a tablet using Redcap software. In addition to questions that captured sociodemographic data, the survey consisted of four questionnaires. Most of these were taken from the cross-NTD Morbidity and Disability Toolkit (https://www.infontd.org/ntd-morbidity-and-disability-nmd-toolkit), which consists of validated tools.

The Washington Group Short Set on Functioning (WG-SS) questionnaire was used as a proxy for disability (WG-SS ≥ 3). This six-item scale asks participants to report limitations in seeing, hearing, walking, remembering/concentrating, caring for themselves, and communicating [23]. The Patient Health Questionnaire (PHQ-9) was used to screen for depression. It is made up of 9 Likert-scale questions, which align with the Diagnostic and Statistical Manual of Mental Disorders criteria for major depressive disorder [24]. The total PHQ-9 score was used to categorise the severity of depression into mild [5–9], moderate [10–14], moderately severe [15–19] or severe [20–27]. The standard cut-off score of ≥10 was used as being indicative of major depressive disorder. The Generalised Anxiety Disorder Questionnaire (GAD-7) was administered to assess anxiety levels. The total score on this seven-item instrument was used to categorise mild [5–9], moderate [10–14] or severe (≥ 15) anxiety. A score of ≥10 was used as a cut-off score for possible generalised anxiety disorder [25]. Finally, the Sari Stigma Scale was used to assess stigma. The scale has 21 items it captures stigma across four domains (experienced, internalised, and anticipated stigma, and disclosure concerns) to arrive at a total stigma score [26].

Questionnaires were translated and back-translated from English into French. Supported by the team, a priest and a teacher from the local communities, they were then further translated between French and Tshiluba, the national language, and back-translated to minimise any discrepancies. The Tshiluba survey was then piloted in the communities before finalisation. A minor adaptation to the PHQ-9 was made to reflect the local context: Item 7, ‘Trouble concentrating on things such as reading newspaper and watching television’ was amended to incorporate more relevant examples - specifically ‘listening to radio or church service’.

#### Key informant interviews.

Eighteen key informants were interviewed to explore their perceptions and experiences of the intervention, in addition to considerations around sustainability and scale-up of the intervention. Informants were purposively selected according to their knowledge and experience of working with persons affected by NTDs, mental health and disability. They included health system stakeholders at the Kasai provincial and Tshikapa zonal levels including representatives of NTD and Leprosy programmes (n = 7), registered nurses (n = 3), community health workers (community relays) (n = 3), community leaders (n = 2), religious leaders (n = 2) and a traditional healer (n = 1). Data collectors approached the key informants through a letter and a follow up telephone call. Each informant was asked to provide informed consent and where relevant consent of the organisation that they were representing (e.g., MoH).

#### Photovoice.

Photovoice was completed with members of the self-help groups and their leaders to document their experience in the delivery and reception of peer support, and on short-term impacts on their mental health and wellbeing. Ten self-help group leaders were asked to take pictures on a) how the peer-support was delivered and b) the impact it had on the lives of participants. In addition, ten members of the groups were purposively selected (based on age and gender). They were asked to take photos of their experiences of being part of the groups and receiving support, with emphasis on the effect it had on their mental health and wellbeing. Individual reflection sessions were conducted with participants to understand the meaning of each photograph. Then, participants grouped photographs into themes and selected three photographs to represent each theme. In group discussions, participants presented their photos and summarised their meanings. Captions were created to describe the photos and were used as data.

#### In-depth interviews.

We conducted an additional five in-depth interviews with group members who did not participate in the photovoice activity to further elucidate participant experience of the self-help group based on the initial themes emerging from the photovoice activities. Participants were purposively selected to represent age, gender, and disease condition. An overview of participants can be found in Table 1.

**Table 1:** Overview of qualitative study participants.

Data collection method	Gender	Ngombe health area		Tshisele health area		Total
		Self-help group leaders	Self-help group members	Self-help group leaders	Self-help group members	
**Photovoice**	Female	3	3	2	2	**10**
	Male	3	3	2	2	**10**
**Sub-total photovoice**						**20**
**In-depth interviews**	Female	0	2	0	1	**3**
	Male	0	1	0	1	**2**
**Sub-total IDIs**						**5**
**Total**		**6**	**9**	**4**	**6**	**25**

### Data analysis

Initially, the quantitative analysis plan was devised around the use of exact pairs of participants that completed both the baseline and the endline survey. However, concerns around reliability of the identification codes used and likely inaccuracies in participant morbidity data from health centres (used at baseline), posed a methodological challenge. Enumerators estimated that around 75% of participants had been surveyed across the two time points. Initial matching based on identification codes and demographic information (age, sex, health zone and disability status) found 51 pairs. Given this partial matching and uncertainty, we decided to treat the dataset as a repeated cross-sectional survey, acknowledging that at least 50% of participants were exactly paired. Importantly, data on self-help group attendance at endline were available and reliable and were used to define the intervention group. However, it was not possible to know which specific individuals at baseline later attended the intervention. As a first step in the analysis process, to facilitate data matching, machine learning was used to predict (based on age, sex, health zone and disability status) which participants in the baseline would have attended the self-help groups. Four different machine learning algorithms were employed: Logistic Regression with Lasso and Ridge regularisation (GLMNET), Random Forest (RF), k-Nearest Neighbors (kNN), and Naive Bayes (NB). Two ensemble models were developed that combined the predictions from the initial base models: generalised linear model (GLM) and a gradient boosting machine (GBM). Further details around the use of these algorithms and model training can be found in S1 File. Data analysis was completed using R software [27].

Descriptive analysis was used to present data on depression, anxiety, and stigma scales, with prevalence rates calculated using recommended cut-off scores where relevant. Reliability of measurement scales was explored using Cronbach’s alpha. A quasi-experimental difference-in-difference (DiD) approach was used to estimate the causal impact of the self-help groups on psychological outcomes (PHQ-9 and GAD-7) and stigma. DiD involves comparing changes in the outcome over time between two groups: a treatment group exposed to the intervention and a control group not exposed to the intervention. The key idea is to observe how the outcome evolves before and after the intervention for both groups and then assess the difference in these changes. The assumption underlying the DiD design is that any difference in the changes observed can be attributed to the intervention, as both groups would have experienced similar trends over time in the absence of the treatment [28]. The DiD was implemented through multiple linear regression analysis, adjusting for the same predictors used in the machine learning prediction section. The interaction term of the variables related to survey period (Time) and attendance of self-help groups (Attendance) provides insight into the impact of the intervention.

Qualitative data was analysed using a thematic framework approach. Interviews were recorded and transcribed verbatim, notes were taken during photo selection and photovoice group discussions and summarised. Transcripts and summaries were translated from French to English and back translated to check overall quality. Using NVivo 12 for windows, we carried out deductive coding using pre-tested topic guides and inductively adjusted as we familiarized ourselves with the data. Four researchers independently coded, charted and mapped emerging themes, two researchers reviewed the analysis process.

### Patient and public involvement

This publication is one outcome from a community-based participatory research study. This means that community members and people with lived experience (peer-researchers) of NTDs and mental health conditions were involved (through participatory workshops) in study design, intervention development and analysis. In addition, peer-researchers were used to recruit participants and undertake data collection. Participants developed a photovoice exhibition to feedback results of the study, and are currently still driving forward the peer-led intervention. Details on their experiences of participating in the study have been reported in a forthcoming paper by Nganda & Seekles, et al [19].

### Ethical approval statement

Ethical approval for this study was obtained from the Congolese National Health Ethics Committee (reference number: 269/CNES/BN/PMMF/2021) and the Liverpool School of Tropical Medicine (reference number: 21–053). Additional information regarding the ethical, cultural, and scientific considerations specific to inclusivity in global research is included in the Supporting Information (S1 Checklist).

## Results

### Survey sample characteristics

A total of 118 persons affected completed the baseline survey; the follow-up survey was completed by 99 persons. Table 2 shows the sample characteristics and mental health outcomes at baseline and endline. The characteristics correspond with those in the synthetic sample (**S1**), indicating accuracy of the machine learning models. The average age of participants was around 50 years at both time points. Men, those living in Tshisele, and those living with a disability constituted the largest proportions in both samples. The self-help groups were attended by 58 respondents. A little over half (n = 31, 53%) of persons affected who participated in the groups were male. The mean age of attendants was 47 (sd 17.3), and 81% were classed as disabled (Table 2).

**Table 2:** Sample characteristics and mental health outcomes.

Characteristic	Baseline (n = 118)	Endline (n = 99)		
	N (%)	Overall N (%)	Self-help group attendance (n = 58)	Self-help group nonattendance (n = 41)
Sex				
Male	73 (61.9)	56 (56.6)	31 (53.4)	25 (61.0)
Female	45 (38.1)	43 (43.4)	27 (46.6)	16 (39.0)
*Age*				
Mean, in years	49.7 (16.7)	49.2 (17.7)	46.8 (17.3)	52.5 (18.0)
*Disability*				
With disability	88 (74.6)	75 (75.8)	47 (81.0)	28 (68.3)
*Depression*				
PHQ-9 mean, SD	13.2 (6.9)	10.9 (5.5)	10.9 (5.6)	10.9 (5.6)
With depression (PHQ-9 ≥ 10)	78 (66.7)	58 (58.6)	37 (63.8)	21 (51.2)
None (0–4)	18 (15.3)	15 (15.2)	10 (17.2)	5 (12.2)
Mild (6–85 –9 )	21 (17.8)	26 (26.3)	11 (19.0)	15 (36.6)
Moderate (11–1310 –14 )	24 (20.3)	37 (37.4)	25 (43.1)	12 (29.3)
Moderately severe (16–1815 –19 )	27 (22.9)	14 (14.1)	9 (15.5)	5 (12.2)
Severe (21–2620 –27 )	27 (22.9)	7 (7.1)	3 (5.2)	5 (12.2)
*Self-harm/Suicidality*	54 (53.8)	64 (35.4)	38 (34.5)	26 (36.6)
*Anxiety*				
GAD-7 mean, SD	10.6 (5.3)	8.8 (3.9)	9.2 (3.9)	8.2 (3.9)
With anxiety (GAD-7 ≥ 10)	68 (57.6)	43 (43.4)	30 (51.7)	13 (31.7)
None (0–4)	22 (18.6)	13 (13.1)	6 (10.3)	7 (17.1)
Mild (6–85 –9 )	28 (23.7)	43 (43.4)	22 (37.9)	21 (51.2)
Moderate (11–1310 –14)	34 (28.8)	36 (36.4)	25 (43.1)	11 (26.8)
Severe (>15)	34 (28.8)	7 (7.1)	5 (8.6)	2 (4.9)
*Stigma*				
Sari mean, SD	39.5 (14.1)	36.3 (14.0)	40.4 (11.7)	30.5 (15.0)

### Mental health outcomes

The mean PHQ-9 score at baseline was 13.2 (sd 6.9) and 66.7% of participants had a score indicative of potential major depressive disorder. In self-help group attendants, the mean endline score was 10.9 (sd 5.6), with 63.8% of participants meeting the major depressive disorder cut-off score. A smaller proportion was classed as severely depressed at endline across the entirety of the person affected population, but particularly amongst those engaged in self-help group activities. In addition, 53.8% of respondents at baseline reported thinking about self-harm or suicide in the two weeks preceding the survey; this was 34.5% in peer support group attendants at endline. Internal consistency of the PHQ-9 was good (α = .79).

The average GAD-7 score was 10.6 (sd 5.3) at baseline, and 57.6% of participants screened positive for general anxiety disorder. For self-help group attendants, at endline, the average score was 9.2 (sd 3.9), with 51.7% being classed as having anxiety and a lower proportion categorised as severely anxious. The Cronbach’s alpha was acceptable (α = 0.68).

Table 3 presents the difference-in-difference regression coefficients for the (identical) ensemble models, with PHQ-9, GAD-7 and Sari as respective outcome variables. Tables with regression coefficients for all models, created using various machine learning algorithms described in the methodology can be found in **S2.** The results show that the support groups significantly impacted on depression and anxiety levels: a significant reduction of PHQ-9 scores was found (ranging on average from -3.5 to -6.7 points, *p* < .05) amongst support group attendants across all models. A 5-point change, which falls within this range, is considered clinically significant. GAD-7 scores also reduced significantly (ranging on average from -2.0 to -3.3 points, *p* < .05) across all models, except for the kNN model, where no statistically significant results were found. These averages are below the minimal clinically important difference of 4 points. Moreover, depression and anxiety were both predicted by having a disability and experiencing higher levels of stigma. Additional 3-way interactions showed that the differences in intervention effect over time did not vary by sex.

**Table 3:** Difference-in-Difference regression coefficients for PHQ-9, GAD-7 and Sari.

	Depression (PHQ-9)	Anxiety (GAD-7)	Stigma (Sari)
Predictor	*b* (SE)	*b* (SE)	*b* (SE)
Time	0.206	-0.003	-5.181
	(1.292)	(0.966)	(2.812)
Attendance	2.100	2.598^**^	4.026
	(1.277)	(0.950)	(2.773)
Time:Attendance	-3.597^*^	-2.499^*^	4.060
	(1.660)	(1.239)	(3.625)
Age	0.008	-0.005	0.023
	(0.025)	(0.019)	(0.055)
Sex ref: male	0.959	0.249	-4.171^*^
	(0.821)	(0.614)	(1.777)
Health zone ref: Tshisele	-1.273	-0.726	3.949
	(0.971)	(0.724)	(2.107)
Disability ref: no	3.250^**^	1.844^*^	9.153^**^
	(0.998)	(0.741)	(2.079)
Stigma	0.138^**^	0.084^**^	
	(0.032)	(0.024)	
Constant	3.629	4.624^**^	29.407^**^
	(2.014)	(1.505)	(3.920)
Observations	216	217	217
R^2^	0.224***	0.201	0.213
Adjusted R^2^	0.195	0.170	0.186
Residual Std. Error	5.800 (df = 207)	4.338 (df = 208)	12.726 (df = 209)
F Statistic	7.490^**^ (df = 8;207)	6.546^**^ (df = 8;208)	8.070^**^ (df = 7;209)

* p < 0.05; ** p < 0.01; ***Coefficients and model fit statistics are based on the full model including all predictors listed.

**Table 4:** Self-Help group components, associated activities and impacts.

Self-Help Group Component	Activities Completed	Evidence of Impact
Self-Care/Wound Management	Capacity strengthening on wound management Provision of self-care kits which included a wash basin, soap Capacity strengthening in individual hygiene and sanitation	“This is a puddle of water on the road. It was wide and is gradually drying out. This is comparable to how my ulcer is healing with the self-care taught by TLM. This practice helps us to care for ulcers effectively. I want other friends who have the same problems to learn and practice self-care. This will greatly improve their mental health” (Photovoice, Person affected, Tshisele, Male, Photo A in S3 File). “It’s a basin, a box of Vaseline and a cup. They trained us on self-care and equipped us with the self-care kits. This gesture had completely calmed my mental health. I have great joy. I always thank God for that. I keep seeing the person I was yesterday and what I have become today” (Photovoice, person affected, female, Tshisele, Photo B in S3 File).
Income Generation Support and Training	Seed funding to start group income generation activity of choice (e.g., soap making, farm cultivation) Support and training on fund management Support on ensuring activities were approved and accepted within the community (e.g., approval of land use for farming)	“This is the image of a cornfield. Corn is the staple meal for us. By selling it, we make financial means. I was a big producer of agricultural products when my vision was good. With my damaged vision, I can no longer produce. It does not mean that I have become weak, I am able to help other people who have the same worries as I had” (Photovoice, person affected, male, Tshisele, Photo D in S3 File) “A photo of the bars of soap. We are happy to have benefited from the training on soap making. We requested this in our BOMOKO group as income generating activity. We can put them on sale and thus have some money. We say thank you to TLM for their support in training and making these soaps. This shows your love for us” (Photovoice, person affected, female, Tshisele, Photo G in S3 File)
Peer-peer counselling and psychoeducation	Group leaders and members shared experiences during meetings Group leaders provided individual counselling sessions with group members Group leaders and members carried out home visits to provide counselling and support to people unable to join the groups. Group leaders and members engaged in fun activities during meetings Mental health awareness session delivered by trained psychologist to self-help groups	“The photo of young amaranths. My brain is like those vegetables that grow on this fertile land. The counselling I received uprooted the bad thoughts that were in my head. Currently my brain is producing good ideas to support others who are suffering with thoughts” (Photovoice, person affected, female, Ngombe, Photo C in S3 File) “These are banana trees together, small and large, bearing bunches of bananas in the process of ripening. This reminds me of the time when we came together with my colleagues who suffer from different NTDs to train as peer helpers in our community where we were marginalised. It was joy for me to find myself among others in this apprenticeship. Today the training has yielded fruits” (Photovoice, person affected, male, Tshisele, Photo E in S3 File) *“*These are pigeons resting together. This photo conveys the calm and unity that we experience during our group meetings. We all love each other despite our different states of health: Lymphatic Filariasis, leprosy, oncho and chronic ulcer. We stay together, we share together and speak for the benefit of all. Wellbeing is everyone’s concern” (Photovoice, person affected, Ngombe, male, Photo F in S3 File)

Qualitative evidence further emphasised the importance of a holistic approach to self-help group structure to improve mental wellbeing. Table 4 provides an overview of key impacts according to key group components, with supporting quotes. Most participants highlighted how the self-care activities were hugely beneficial. They identified improved mobility, better wound management (e.g., reduction in ulcer/wound size), and a reduction in visible changes to the skin as a result of the self-care components of the intervention. Participants clearly linked their physical health to their mental health, stating that self-care improved or calmed their mental health. Livelihoods training and support from the study team enabled the groups to set up income generating activities. In Ngombe, the group opted for soap making, whilst in Tshisele farming provided income for group members. These activities reminded participants of their value and enable them to develop a sense of self-awareness. They also led some group members to recognise their economic potential. For example, a photovoice participant who benefitted from soap making hopes to expand the group’s income generating activities:

*It’s a home-grown cassava field. This field is cultivated by one person but nourished many others. When its production is sold, it brings money to the family. It’s the same with us in the BOMOKO group: today with the production and sale of soap, we hope to have a profit that can help us solve small daily problems. We further hope that in time we will do even more than this, like grow cassava. (Photovoice, Person affected, Ngombe, Male, Photo H in* *S3 File* *)*

Finally, many persons affected described a sense of relief from negative thoughts and feelings, embracing positivity in their lives as a result of peer-peer support and counselling as well as the psychoeducation provided which enabled them to understand and explore feelings they were having. They felt included and happy sharing their experiences in this common space, which they perceived as beneficial. Together, all components of the self-help groups enabled people to find peace and tranquillity with a holistic focus on their wellbeing.

### Community participation and stigma

The survey results did not show an impact of the intervention on overall stigma levels (Table 3) and stigma domain scores (e.g., internalised, externalised stigma). The total stigma score was 39.5 (sd 14.1) at baseline and 40.4 (sd 11.7) at endline for support group attendants (Table 2). However, qualitative findings presented a slightly different picture, with many respondents reflecting on improvements to their self-esteem and ability to take part in community life. Most persons affected described re-engaging with community and family activities including, visiting the market, attending church and funerals. Community members and health professionals also observed these behavioural changes and perceived a reduction in experiences of stigma and discrimination for persons affected. Self-help groups were identified as safe spaces that gave people a sense of belonging, unity, reassurance, and hope.

*“The market day, for example in NGOMBE, is twice a week. Before, it was difficult to see a person affected coming to the market, looking for supplies. But now they integrate without feeling embarrassed, they come with their little means to purchase an item or a need. Even at the health center they now have easy access. Stigma against the people we have supported is noticeably going away” (KII, health professional, Tshikapa, male)*

**Fig 2:**
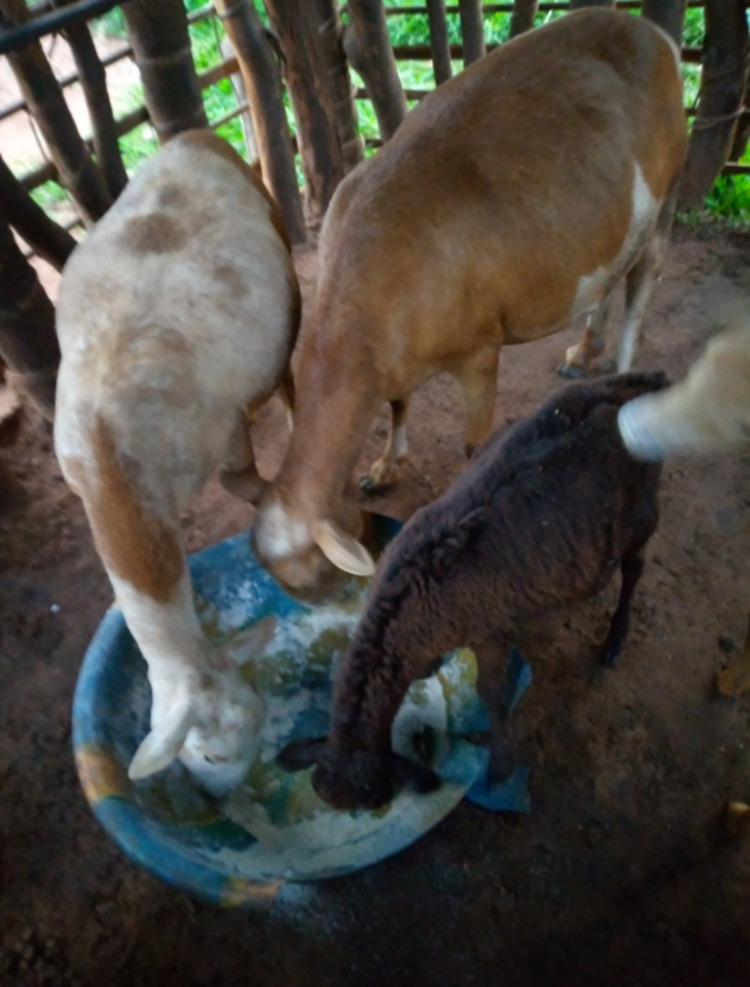
Goats.

*“These are sheep eating together in a stable. I am very satisfied today to eat with others. Before TLM I didn’t have the courage to do it. Currently, I eat with the other members of my family. We share with the community without difficulty” (* *Fig 2* *, Photovoice, person affected, Ngombe, female)*

**Fig 3:**
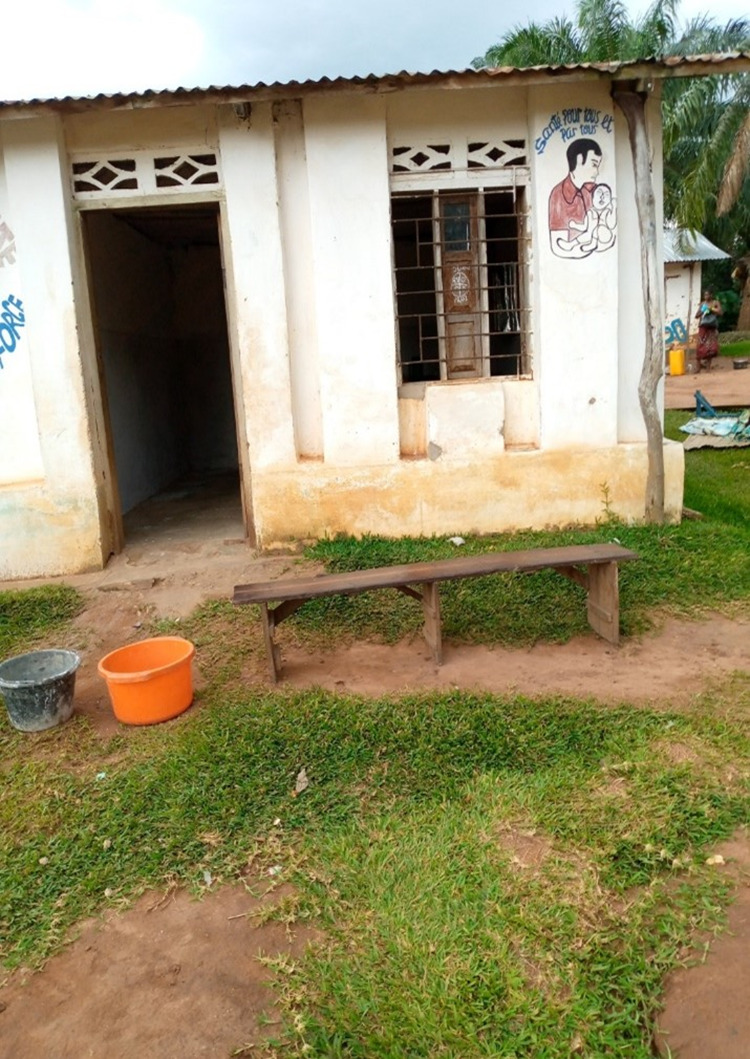
Neighbourhood health centre.

*“A photo of a neighborhood health center. In the past, I had difficulty being accepted at the health centre. It was since I was provided with the TLM self-care support that the partners connected me with the health center. Today I am proud to be in communication with community relays” (* *Fig 3* *, Photovoice, person affected, Ngombe, male)*

## Discussion

As a community-based, peer-led mental health intervention, ‘*Peace of Mind’* combined lay counselling, mutual support, self-care, and income generation activities within a self-help group model to address health, psychosocial and economic impacts of skin-NTDs. The findings showed that this holistic approach was effective at reducing the prevalence of common mental health conditions (depression and anxiety) in affected persons, supporting WHO recommendations that advocate for such initiatives [18]. These results were found after an implementation period of six months, which shows that improvements can be made over a relatively short period of time, as also found by Dellar et al who reported reduced levels of depression after three months of a similar intervention [17].

To our knowledge, this was one of the first studies to quantitatively assess the impact of support groups on mental health of persons affected by NTDs; two larger trials on similar interventions are currently underway [29,30]. Whilst this limits opportunities for comparison with NTD-related studies, the current findings are in line with evidence, mainly from high income countries, of positive outcomes of support groups for people with common mental health conditions more generally [31,32]. Existing NTD studies have also reported reduced depression scores, and highlighted broader beneficial impacts of self-help groups on stigma [33], community cohesion, social participation, livelihood activities and self-care practices [13].

It is important to note that even though levels of depression and anxiety reduced, they remained elevated, with a substantial proportion of self-help group attendants scoring above clinical cut-off scores. In addition, suicidal/self-harm thoughts were still reported by 35% of respondents. Therefore, our findings show that whilst holistic support groups have the potential to serve as a key component of integrated NTD/mental health service provision at community-level, this requires time and further input at primary health care, and the establishment of mental health services at secondary and tertiary care levels is necessary to adequately support the biopsychosocial needs of persons affected by NTDs in the region. A Congolese psychologist on the study team facilitated a mental health session during one of the group meetings and offered pathways for voluntary self-referral; however, no participants engaged with this option. This lack of uptake points to potential demand-side barriers—such as stigma or limited mental health literacy—that merit further investigation. The current intervention did not specifically aim to reduce stigma and we were only able to identify impacts on stigma qualitatively. Adding a focused stigma-reduction component to the intervention, such as a rights-based counselling approach and other contact-based interventions alongside increased advocacy and awareness campaigns, might lead to a greater improvement in mental health outcomes [34].

Whilst it was not possible to quantify the influential properties of each separate intervention component on mental health outcomes, as a strength, the mixed-method approach of this study allowed for in-depth qualitative explorations of intervention impact. These support theoretical underpinnings of our intervention that propose that improvements in mental health outcomes stem directly from the mental health components of the intervention, but also from broader impacts related to opportunities for self-care, income generation, and reduced internalised stigma linked to increased group-based identity. Our study corroborates the findings of Jay et al in Nepal, which emphasise a critical impact of self-help groups and the positive impact they have on feelings of belonging and shared identity [35].

This study was limited by a relatively small sample size (due to a finite number of hard-to-reach persons affected by NTDs in the study area) and difficulties with obtaining a matched baseline and endline sample. Because of this, we could not complete an analysis based on exact pairs, which would have added further rigour. Specifically, it would have also allowed us to understand if there were differences in baseline scores for those patients who selected themselves to be part of the intervention and those who did not. However, machine learning techniques in combination with the DiD design still allowed us to estimate the causal impact of the intervention, enhancing the internal validity of the study. This quasi-experimental design was a significant strength of the study and we do believe that we have demonstrated the potential of this intervention. Future research should also consider the appropriateness and effectiveness of the intervention across multiple NTD types and could explore difference in impact between group leaders and attendants. In the current study, the decision was made to include all persons affected regardless of the type of skin NTD they were affected by, since synergies were expected in the biopsychosocial impact of the diseases [4]. However, concerns around the reliability of health facility data on disease type that was supposed to be linked to survey data, meant that the quantitative analysis could not explore any differences in intervention impact across different NTDs. Furthermore, while this impact evaluation included a measure of disability, we did not apply a formal morbidity grading system. Including such measures could have provided a more nuanced understanding of the relationship between the severity of physical impairments and the outcomes of self-help group participation.

Finally, future research should explore opportunities for scale-up and long-term impact and sustainability of the intervention. A forthcoming paper on the implementation process of the current intervention is likely to be a useful resource in the preparatory phase of such a study. To date, early insights from the continuation phase of the intervention suggest promising feasibility for long-term sustainability. With support from TLM and Effect Hope, the groups have sustained regular meetings and income-generating activities. We believe that our community-led, participatory approach to intervention development and delivery was a key driver in sustainability, fostering local ownership and leveraging existing structures (such as support from community health workers). To support the groups on their path to independence, there has been a deliberate investment in governance and advocacy structures during the continuation phase. Core groups have established inclusive leadership committees, received training in rights-based lobbying and advocacy to equip members with the skills and confidence to engage with local authorities and seek partnerships with other organisations for Persons with Disabilities. The groups are progressing towards formal legal recognition. To support long-term sustainability, a community fund will be launched, which allows for covering operational needs and supporting micro-loans for individual members, indicating a scalable model of financial resilience. Importantly, to ensure that income generation does not overshadow the foundational psychosocial elements, refresher BPS-N and training-of-trainers training has been provided, to build a pipeline of new peer helpers. Groups have continued to set aside a portion of each meeting for peer support and are encouraged to continue to do so. Monitoring and evaluation tools have been co-developed with group members, to track peer leader performance, physical and mental health support, in addition to the group’s financial status. Individual level tools (including psychosocial indicators) have also been developed to guide peer leaders in conducting regular, structured check-ins on members’ physical and mental health.

## Supporting information

S1 File Machine Learning Process and Predictions. (DOCX)

S2 File Difference-in-Difference regression models for all machine learning models for PHQ-9, GAD-7 and SARI. (DOCX)

S3 File Illustrative Photos from Photovoice Activity. (DOCX)

S4 File Endline minimal dataset. (CSV)

S5 File Coding framework for Endline analysis. (DOCX)

S1 Checklist Inclusivity in global research. (DOCX)
