# Supplementary material for: Peace of mind: A quasi-experimental, mixed-method evaluation of a community-based mental health intervention for persons affected by Neglected Tropical Diseases
Source: PLOS Ment Health. 2025 Sep 4;2(9):e0000423. doi: 10.1371/journal.pmen.0000423 (PMC12798642; doi:10.1371/journal.pmen.0000423)
Supplement: S1 File — (DOCX) [file pmen.0000423.s001.docx]

**Supplementary File 1: Machine Learning process and predictions**

Machine learning models were used to predict which baseline participants would or would not have joined the peer support groups, for matching purposes. We evaluated four different machine learning algorithms for predicting event attendance: Logistic Regression with Lasso and Ridge regularization (GLMNET), Random Forest (RF), k-Nearest Neighbors (kNN), and Naive Bayes (NB). We then developed two ensemble models that combines the predictions from the base models: generalised linear model and a gradient boosting machine.

We performed model training using the `caret` package in R. For each model, we conducted hyperparameter tuning using repeated cross-validation with 20 folds to ensure robustness and avoid overfitting.

We trained each model on the training dataset and evaluated their performance on the test dataset. The evaluation metrics used the area under the receiver operating characteristic curve (AUC-ROC). We also generated confusion matrices to assess the models' performance in predicting event attendance.

Table 1 shows the median evaluation metrics (Receiver Operating Characteristic curve, Sensitivity and Specificity) for the models used. The sensitivity median values range from approximately 0.00 to 1.00 for the individual models and ensemble models. The specificity median values range from approximately 0.67 to 1.00 for the individual models and ensemble models. The median values were highest for the ensemble models (e-GLM/e-GBM), indicating their increased predictive performance in terms of ROC, Sensitivity and Specificity over the individual models in the context of predicting those who attended the peer support groups and those who did not.

Table 1 Median Evaluation Metrics for machine learning models

| **Model** | **ROC** | **Sensitivity** | **Specificity** |
| --- | --- | --- | --- |
| GLMNET | 0.67 | 0.50 | 0.67 |
| RF | 0.67 | 0.50 | 0.67 |
| SVM | 0.67 | 0.00 | 1.00 |
| kNN | 0.54 | 0.50 | 0.67 |
| NB | 0.67 | 0.50 | 0.67 |
| e-GLM | 1.00 | 1.00 | 1.00 |
| e-GBM | 0.81 | 0.75 | 1.00 |

Table 2 presents the characteristics of participants in the synthetic sample at baseline and endline. These correspond with those of the ‘true’ sample (Table 2 in paper), indicating accuracy of the machine learning models.

Table 2 Synthetic sample characteristics

| **Characteristic** | **Baseline**  **N = 118** | **Endline**  **N = 99** |
| --- | --- | --- |
| *Age*, average (SD) | 50 (17) | 50 (18) |
| *Sex* |  |  |
| Male | 73 (62%) | 56 (57%) |
| Female | 45 (38%) | 43 (43%) |
| *Health zone* |  |  |
| Tshisele | 84 (71%) | 71 (72%) |
| Ngombe | 34 (29%) | 28 (28%) |
| *Disability* |  |  |
| Without | 30 (25%) | 24 (24%) |
| With | 88 (75%) | 75 (76%) |
