# Supplementary material for: Peace of mind: A quasi-experimental, mixed-method evaluation of a community-based mental health intervention for persons affected by Neglected Tropical Diseases
Source: PLOS Ment Health. 2025 Sep 4;2(9):e0000423. doi: 10.1371/journal.pmen.0000423 (PMC12798642; doi:10.1371/journal.pmen.0000423)
Supplement: S2 File — (DOCX) [file pmen.0000423.s002.docx]

**S2: Difference-in-Difference regression models for all machine learning models for PHQ-9, GAD-7 and SARI**

Table 1 Diff-in-Diff regression coefficients PHQ-9

| *Outcome: PHQ-9* | **GLMNET** | **RF** | **kNN** | **NB** | | **e-GLM/**  **e-GBM** |
| --- | --- | --- | --- | --- | --- | --- |
| **Predictor** | ***b* (SE)** | ***b* (SE)** | ***b* (SE)** | ***b* (SE)** | | ***b* (SE)** |
| Time | 2.170^*^ | 0.206 | 0.203 | 0.429 | | 0.206 |
|  | (1.257) | (1.292) | (1.341) | (1.187) | | (1.292) |
|  |  |  |  |  | |  |
| Attendance | 5.899^***^ | 2.100 | 1.874 | 3.838^***^ | | 2.100 |
|  | (1.316) | (1.277) | (1.283) | (1.394) | | (1.277) |
|  |  |  |  |  | |  |
| Time:Attendance | -6.725^***^ | -3.597^**^ | -3.489^**^ | -4.837^***^ | | -3.597^**^ |
|  | (1.624) | (1.660) | (1.706) | (1.654) | | (1.660) |
|  |  |  |  |  | |  |
| Age | 0.027 | 0.008 | 0.011 | 0.019 | | 0.008 |
|  | (0.024) | (0.025) | (0.026) | (0.025) | | (0.025) |
|  |  |  |  |  | |  |
| Sex ref: male | 0.129 | 0.959 | 0.815 | 0.420 | | 0.959 |
|  | (0.811) | (0.821) | (0.829) | (0.836) | | (0.821) |
|  |  |  |  |  | |  |
| Health zone ref: Tshisele | -2.164^**^ | -1.273 | -0.805 | -2.074^**^ | | -1.273 |
|  | (0.940) | (0.971) | (0.926) | (1.027) | | (0.971) |
|  |  |  |  |  | |  |
| Disability ref: no | 1.950^*^ | 3.250^***^ | 3.408^***^ | 2.983^***^ | | 3.250^***^ |
|  | (1.009) | (0.998) | (0.992) | (0.994) | | (0.998) |
|  |  |  |  |  | |  |
| Stigma | 0.129^***^ | 0.138^***^ | 0.138^***^ | 0.124^***^ | | 0.138^***^ |
|  | (0.030) | (0.032) | (0.032) | (0.032) | | (0.032) |
|  |  |  |  |  | |  |
| Constant | 2.207 | 3.629^*^ | 3.382 | 3.710^**^ | | 3.629^*^ |
|  | (1.845) | (2.014) | (2.159) | (1.848) | | (2.014) |
|  | | | | | | |
| Observations | 216 | 216 | 216 | | 216 | 216 |
| R^2^ | 0.284 | 0.224 | 0.222 | | 0.242 | 0.224 |
| Adjusted R^2^ | 0.256 | 0.195 | 0.192 | | 0.213 | 0.195 |
| Residual Std. Error | 5.574  (df = 207) | 5.800  (df = 207) | 5.808  (df = 207) | | 5.734  (df = 207) | 5.800  (df = 207) |
| F Statistic | 10.255^***^  (df = 8; 207) | 7.490^***^  (df=8; 207) | 7.399^***^  (df = 8; 207) | | 8.266^***^  (df = 8; 207) | 7.490^***^  (df = 8; 207) |

Table 2 Diff-in-Diff regression coefficients GAD-7

| *Outcome: GAD-7* | **GLMNET** | **RF** | **kNN** | **NB** | **e-GLM/**  **e-GBM** |
| --- | --- | --- | --- | --- | --- |
| **Predictor** | ***b* (SE)** | ***b* (SE)** | ***b* (SE)** | ***b* (SE)** | ***b* (SE)** |
| Time | 0.474 | -0.003 | -0.269 | -0.611 | -0.003 |
|  | (0.963) | (0.966) | (1.010) | (0.901) | (0.966) |
|  |  |  |  |  |  |
| Attendance | 3.622^***^ | 2.598^***^ | 1.892^*^ | 2.297^**^ | 2.598^***^ |
|  | (1.008) | (0.950) | (0.964) | (1.059) | (0.950) |
|  |  |  |  |  |  |
| Time:Attendance | -3.285^***^ | -2.499^**^ | -1.976 | -2.079^*^ | -2.499^**^ |
|  | (1.246) | (1.239) | (1.284) | (1.257) | (1.239) |
|  |  |  |  |  |  |
| Age | -0.001 | -0.005 | -0.005 | -0.007 | -0.005 |
|  | (0.018) | (0.019) | (0.020) | (0.019) | (0.019) |
|  |  |  |  |  |  |
| Sex ref:male | -0.269 | 0.249 | 0.103 | -0.078 | 0.249 |
|  | (0.623) | (0.614) | (0.624) | (0.636) | (0.614) |
|  |  |  |  |  |  |
| Health zone ref:Tshisele | -0.985 | -0.726 | -0.146 | -0.906 | -0.726 |
|  | (0.722) | (0.724) | (0.697) | (0.782) | (0.724) |
|  |  |  |  |  |  |
| Disability ref:without | 1.126 | 1.844^**^ | 2.039^***^ | 1.779^**^ | 1.844^**^ |
|  | (0.773) | (0.741) | (0.742) | (0.753) | (0.741) |
|  |  |  |  |  |  |
| Stigma | 0.082^***^ | 0.084^***^ | 0.085^***^ | 0.079^***^ | 0.084^***^ |
|  | (0.023) | (0.024) | (0.024) | (0.024) | (0.024) |
|  |  |  |  |  |  |
| Constant | 4.744^***^ | 4.624^***^ | 4.730^***^ | 5.674^***^ | 4.624^***^ |
|  | (1.410) | (1.505) | (1.626) | (1.401) | (1.505) |
|  | | | | | |
| Observations | 217 | 217 | 217 | 217 | 217 |
| R^2^ | 0.221 | 0.201 | 0.187 | 0.191 | 0.201 |
| Adjusted R^2^ | 0.191 | 0.170 | 0.156 | 0.160 | 0.170 |
| Residual Std. Error | 4.284  (df = 208) | 4.338  (df= 208) | 4.375  (df= 208) | 4.366  (df = 208) | 4.338  (df = 208) |
| F Statistic | 7.366^***^  (df=8; 208) | 6.546^***^  (df=8;208) | 5.998^***^ (df=8;208) | 6.128^***^ (df=8; 208) | 6.546^***^  (df =8;208) |

Table 3 Diff-in-Diff regression coefficients Sari

| *Outcome: Sari* | **GLMNET** | **RF** | **kNN** | **NB** | **e-GLM/**  **e-GBM** |
| --- | --- | --- | --- | --- | --- |
| **Predictor** | ***b* (SE)** | ***b* (SE)** | ***b* (SE)** | ***b* (SE)** | ***b* (SE)** |
| Time | -5.147^*^ | -5.181^*^ | -4.413 | -3.807 | -5.181^*^ |
|  | (2.838) | (2.812) | (2.916) | (2.566) | (2.812) |
|  |  |  |  |  |  |
| Attendance | 4.267 | 4.026 | 4.822^*^ | 9.386^***^ | 4.026 |
|  | (2.981) | (2.773) | (2.779) | (2.959) | (2.773) |
|  |  |  |  |  |  |
| Time:Attendance | 3.971 | 4.060 | 3.117 | -0.194 | 4.060 |
|  | (3.692) | (3.625) | (3.720) | (3.598) | (3.625) |
|  |  |  |  |  |  |
| Age | 0.020 | 0.023 | 0.039 | 0.057 | 0.023 |
|  | (0.055) | (0.055) | (0.058) | (0.055) | (0.055) |
|  |  |  |  |  |  |
| Sex ref:male | -4.779^***^ | -4.171^**^ | -4.533^**^ | -5.372^***^ | -4.171^**^ |
|  | (1.822) | (1.777) | (1.783) | (1.783) | (1.777) |
|  |  |  |  |  |  |
| Health zone ref:Tshisele | 3.867^*^ | 3.949^*^ | 4.811^**^ | 1.525 | 3.949^*^ |
|  | (2.130) | (2.107) | (1.994) | (2.234) | (2.107) |
|  |  |  |  |  |  |
| Disability ref:without | 8.395^***^ | 9.153^***^ | 9.383^***^ | 7.964^***^ | 9.153^***^ |
|  | (2.222) | (2.079) | (2.055) | (2.083) | (2.079) |
|  |  |  |  |  |  |
| Constant | 30.257^***^ | 29.407^***^ | 27.659^***^ | 27.749^***^ | 29.407^***^ |
|  | (3.630) | (3.920) | (4.314) | (3.520) | (3.920) |
|  |  |  |  |  |  |
|  | | | | | |
| Observations | 217 | 217 | 217 | 217 | 217 |
| R^2^ | 0.213 | 0.213 | 0.216 | 0.241 | 0.213 |
| Adjusted R^2^ | 0.186 | 0.186 | 0.190 | 0.216 | 0.186 |
| Residual Std. Error | 12.728  (df = 209) | 12.726  (df = 209) | 12.699  (df = 209) | 12.493  (df = 209) | 12.726  (df = 209) |
| F Statistic | 8.059^***^  (df = 7; 209) | 8.070^***^  (df=7; 209) | 8.232^***^  (df=7; 209) | 9.499^***^  (d=7;209) | 8.070^***^  (df=7;209) |
