# Supplementary material for: Peace of mind: A quasi-experimental, mixed-method evaluation of a community-based mental health intervention for persons affected by Neglected Tropical Diseases
Source: PLOS Ment Health. 2025 Sep 4;2(9):e0000423. doi: 10.1371/journal.pmen.0000423 (PMC12798642; doi:10.1371/journal.pmen.0000423)
Supplement: S3 File — (DOCX) [file pmen.0000423.s003.docx]

**S3 File: Illustrative Photos from Photovoice Activity**


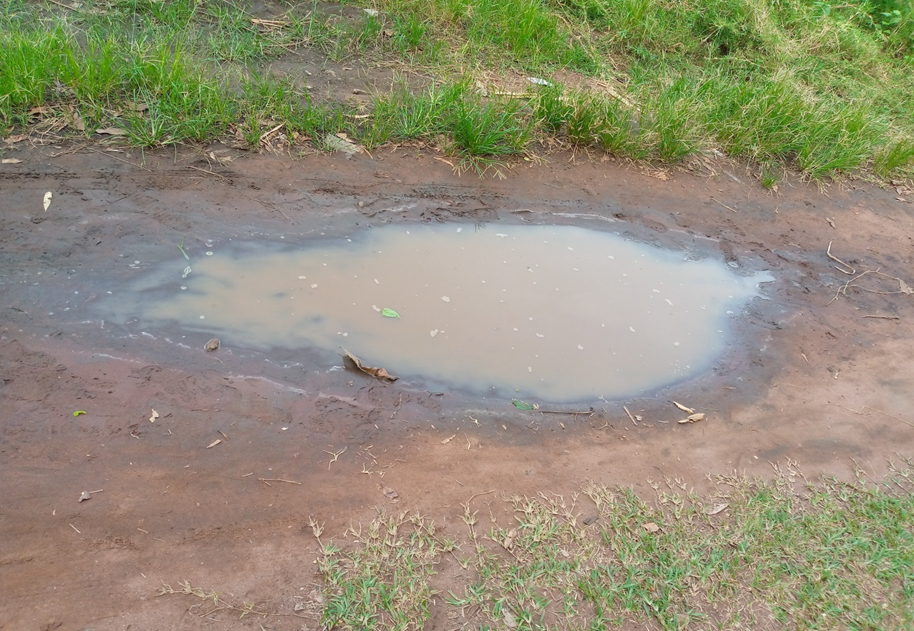


Photo A


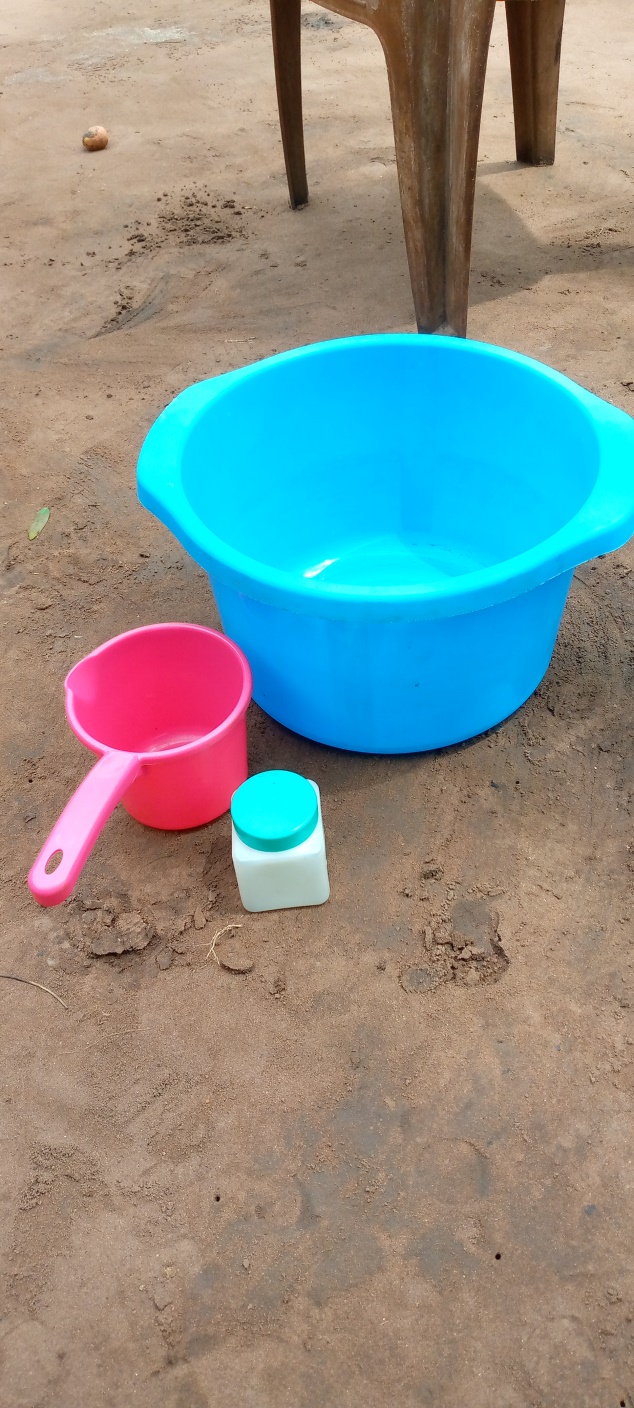


Photo B


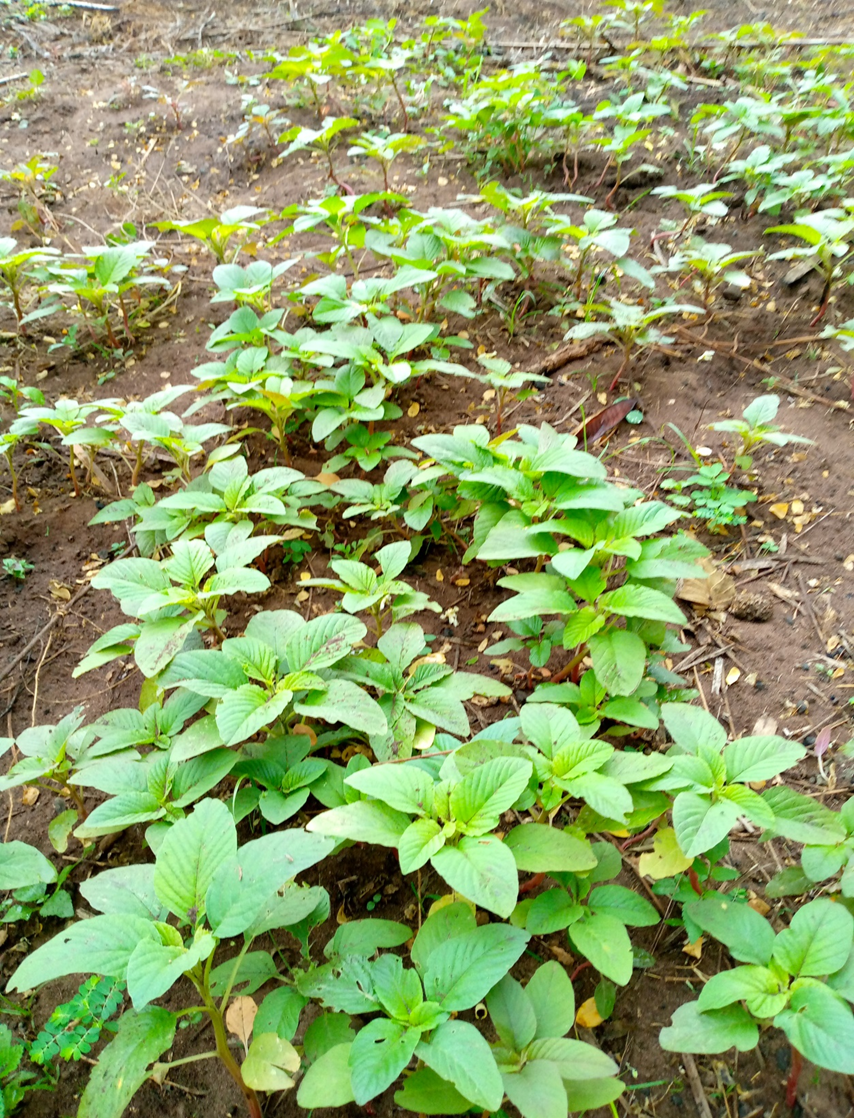


Photo C


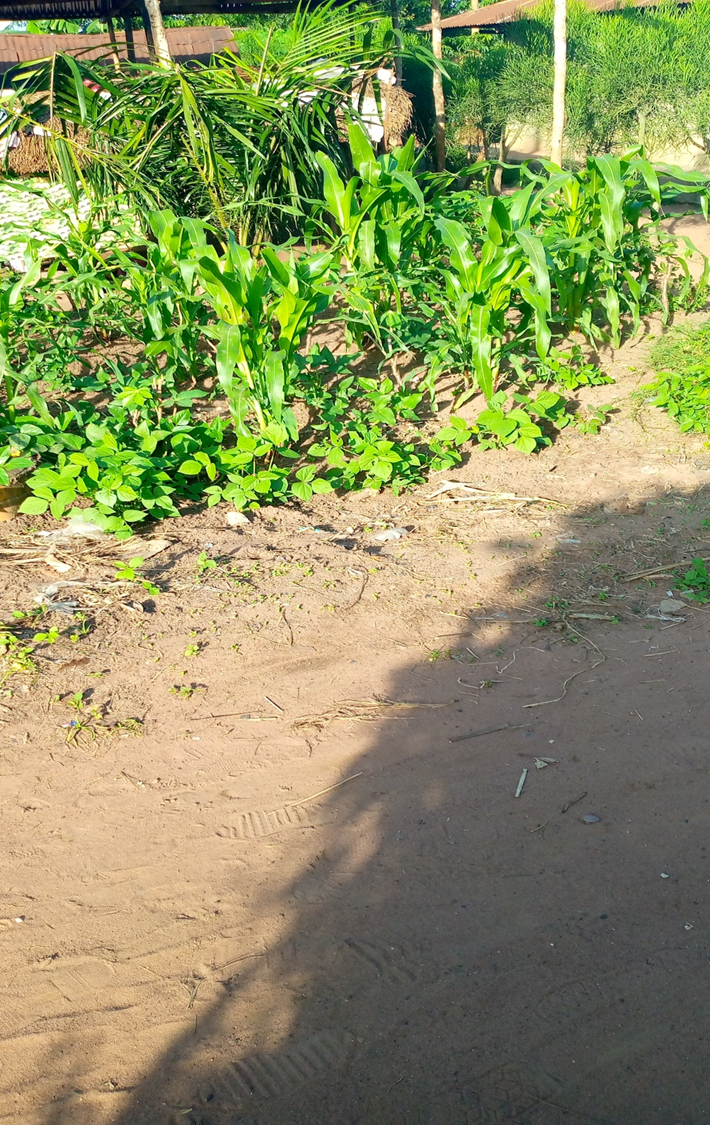


Photo D


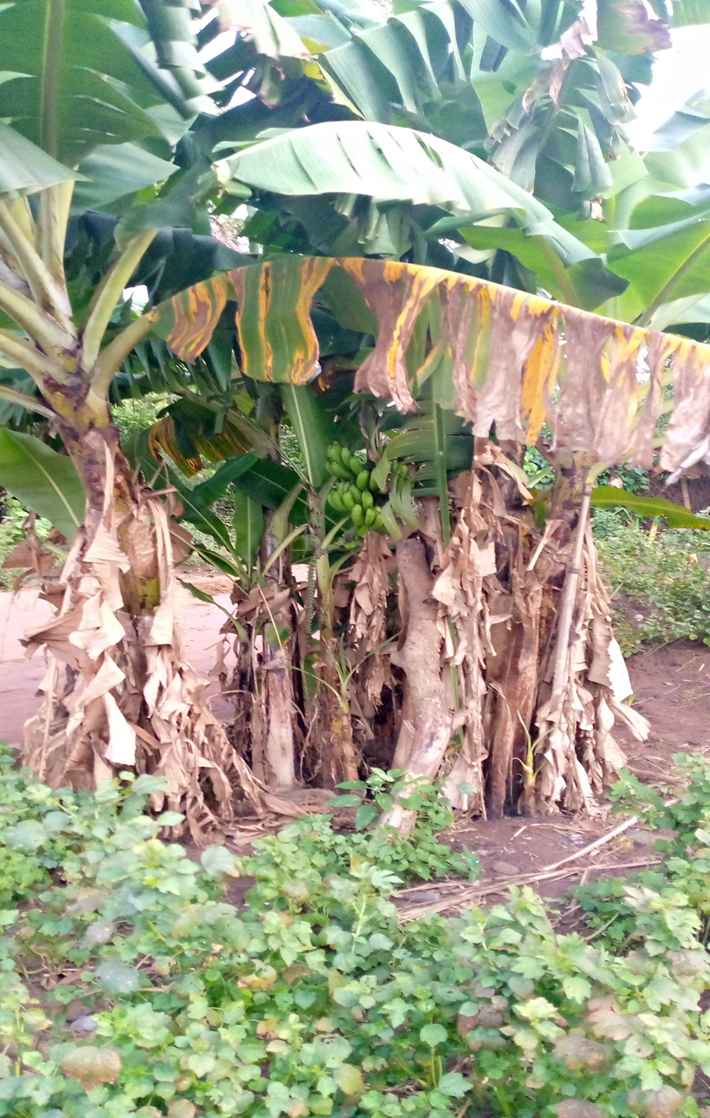


Photo E


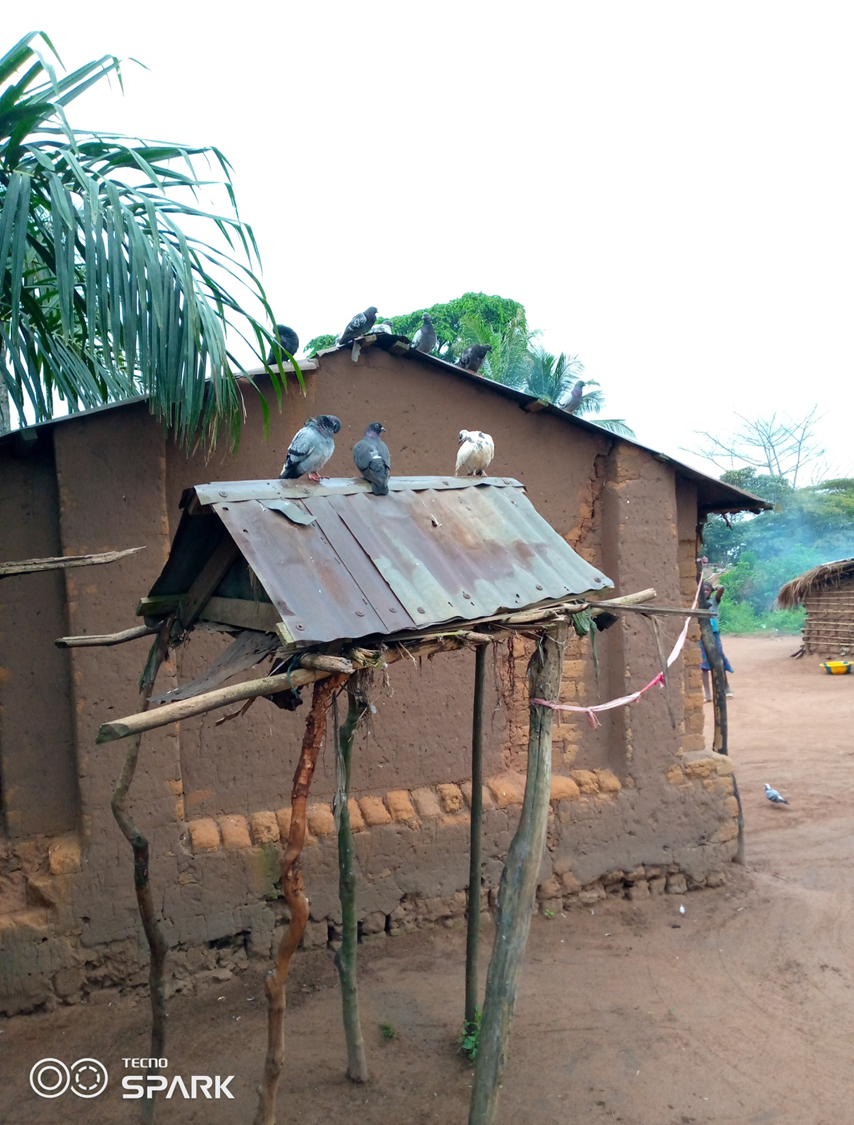


Photo F


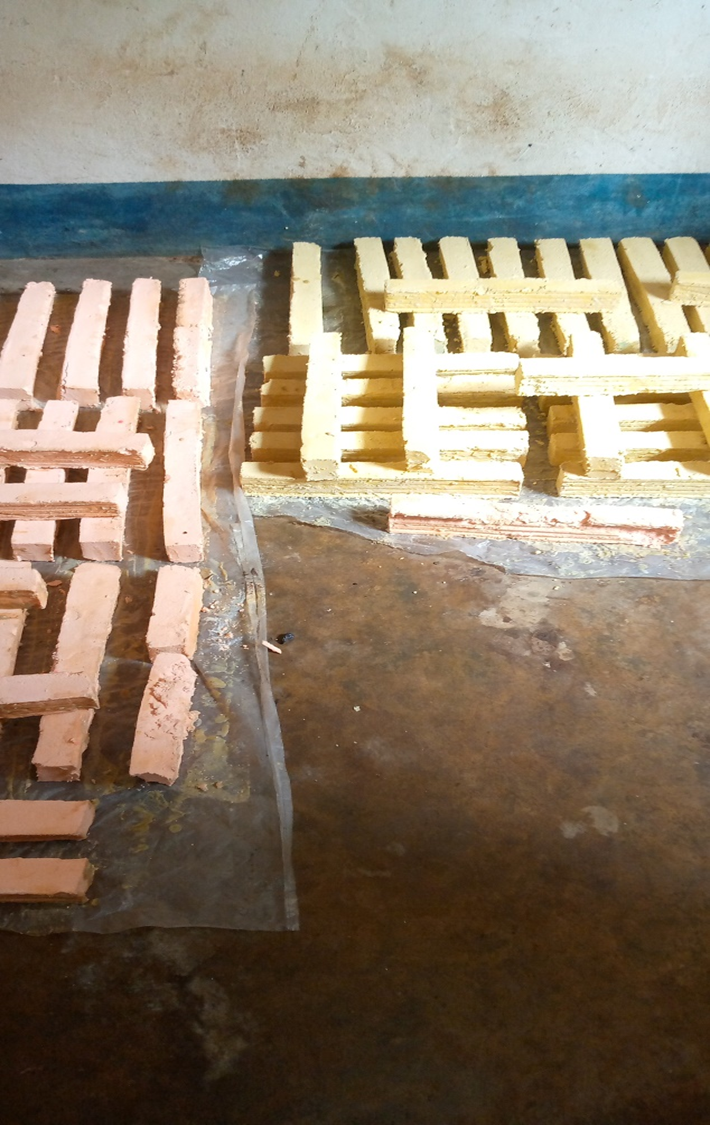


Photo G


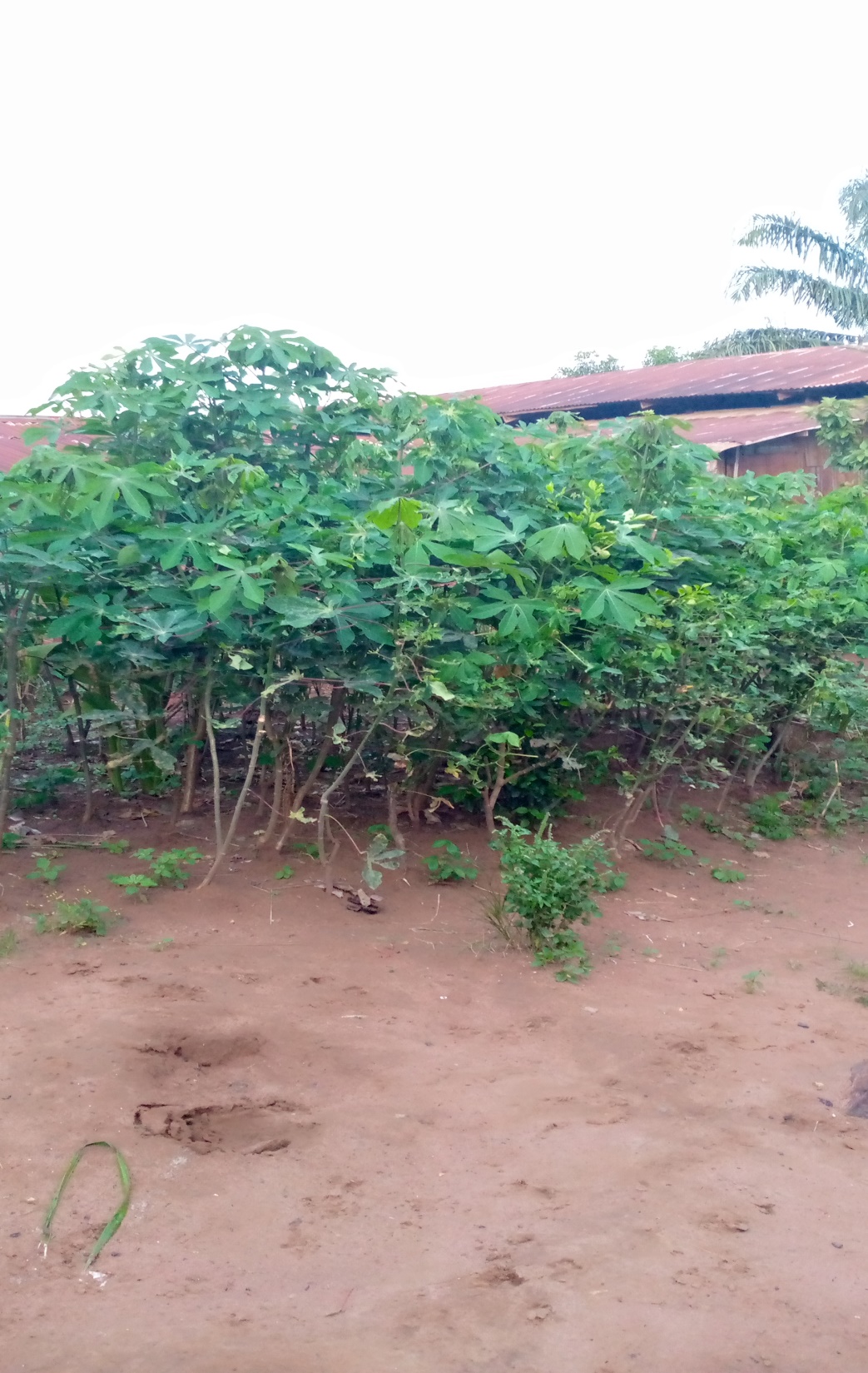


Photo H
