# Supplementary material for: Peace of mind: A quasi-experimental, mixed-method evaluation of a community-based mental health intervention for persons affected by Neglected Tropical Diseases
Source: PLOS Ment Health. 2025 Sep 4;2(9):e0000423. doi: 10.1371/journal.pmen.0000423 (PMC12798642; doi:10.1371/journal.pmen.0000423)
Supplement: S5 File — (DOCX) [file pmen.0000423.s005.docx]

KDDM study Endline Analysis

Coding framework for qualitative data

1. Demography (of participants)

- Identifier
- Date of interview
- Age
- Sex
- Profession / job role
- Longevity of service
- Residence

1. Experience working with people living with chronic health conditions

- Experience with people with skin NTDs
- Experience with people with other chronic health conditions

1. Challenges faced by people affected by skin NTDs

- Stigma
- Isolation
- Lack of community integration
- Addiction problems
- Lack of sources of revenue
- Dependence on other people for daily living
- Discrimination by family members
- Called derogatory names by family members and community
- Persistent pain and discomfort
- Loss of livelihoods

1. Challenges faced by participants to support people affected by skin NTDs

- Long-distance travel to hard-to-reach areas where people affected live

1. Participants’ involvement in the design and development of the MH intervention

- Active participation in meetings
- Engagement with the community advisory board

1. Participants’ contribution to the design and development of the MH intervention

- Input ideas during meetings and workshops from personal experience

1. Participants’ contribution to the implementation of the MH intervention
2. Most important/interesting aspects of the intervention

- The photo dissemination workshop
  - Inclusivity
  - Freedom of speech
  - PV participants explaining their photos and expressing their feelings through their photos
- The intervention development
- The implementation processes
  - Follow-up of people affected in their communities

1. Actors involved in the design and development of the MH intervention

- Attribution of actors
  - People living with skin NTDs
  - Health workers
  - Community leaders
  - Community relays
  - Faith based healers
  - Political and administrative authorities
- Recruitment of actors
  - Random selection from the community
  - Able to convey learnings to the community
- Strengthening collaboration with actors
  - All-inclusive approach

1. What worked well during the design and development of the MH intervention

- The meetings
- Follow up on the plans agreed on during the meetings

1. Challenges faced in the design and development of the intervention
2. How MH intervention was organized and why
3. Effectiveness of the MH intervention

- Counselling
- Selfcare training and provision of selfcare kits

1. Focus of the MH intervention

- Mental illness
- Mental health
- NTDs

1. Needs for people affected by skin NTDs identified during the intervention

- Care needs
- Wellbeing needs

1. How needs were identified

- During workshop discussions

1. What went well during the implementation of the MH intervention

- Follow up of people affected by skin NTDs
- The soap making process
- The friendly approach of the selfcare training sessions
- Counselling sessions on courage and communication during peer-support group meetings

1. Challenges faced in the implementation of the MH intervention

- One type of seed was supplied, and supplied late for the season

1. Number and composition of participants of the MH study

- Number of people who participated in the intervention
- Composition of the participants

1. Supervision of the intervention

- How supervision was organized
  - Terms of references and mission orders
  - Action plans
  - Frequency of supervision
- Supervisors
- Who were supervised
- What the supervision was on
- Facilitators of the supervision process
- Challenges during supervision

1. Activities carried out during peer-support groups

- Counselling sessions on courage and communication
- Selfcare
- Decision-making discussions e.g. on group finances
- Home visits
- Soap making

1. Contribution of participants in peer support groups

- Facilitator on selfcare after haven received training
- Home visits

1. Impact of the MH intervention (Impact of the peer-support groups)

- On people affected by skin NTDs
  - Shared space for people affected
  - Improved peace of mind
  - Decreased suicidal / self-harming thoughts
  - Improved community integration
  - Improved communication with community members
  - Improved knowledge on skin care
  - Improved overall physical health e.g. participants able to walk following continues care
  - Regained a source of revenue e.g. selling soap
  - Improved knowledge on selfcare
  - Benefitted from selfcare kits
- On the community
  - Reduced discrimination
  - Community integration with people affected by skin NTDs
- On the participant
  - Emerging system of entrepreneurship with people affected may ease work on self-sufficiency
  - Soap manufactured locally may improve self-care
- Extend to which intervention addressed needs
  - Intervention addressed discrimination against persons affected
- Strengths of the intervention
  - Self-care training and provision of self-care kits
  - Follow-up of persons affected
- Weaknesses of the intervention
  - Sustainability of intervention is uncertain
  - Intervention still dependent on external funding and the state
  - Supply chain of self-care inputs is not constant

1. How intervention can be improved

- Include construction of portable water to support people affected with skin care
- Include distribution of medications in the intervention, such as pain killers

1. Sustainability of the MH intervention

- Maintain planned follow-up of people living with skin NTDs
- Include young leaders of the community in peer-support groups
- Increase community sensitization on skins NTDs, persons affected and on the MH intervention
- Scale up to other communities through sensitization
- Maintain activities of the peer-support groups: counselling, home visits, soap making, etc.

1. External factors that may have influenced intervention outcomes
